# The impact of CdSe/ZnS Quantum Dots in cells of *Medicago sativa *in suspension culture

**DOI:** 10.1186/1477-3155-8-24

**Published:** 2010-10-07

**Authors:** Ana R Santos, Ana S Miguel, Leonor Tomaz, Rui Malhó, Christopher Maycock, Maria C Vaz Patto, Pedro Fevereiro, Abel Oliva

**Affiliations:** 1 Biomolecular Diagnostics Laboratory, Instituto de Tecnologia Química e Biológica, Universidade Nova de Lisboa, Apartado 127, 2781-901 Oeiras, Portugal; 2 Plant Cell Biotechnology Laboratory, Instituto de Tecnologia Química e Biológica, Universidade Nova de Lisboa, Apartado 127, 2781-901 Oeiras, Portugal; 3 Organic Synthesis Laboratory, Instituto de Tecnologia Química e Biológica, Universidade Nova de Lisboa, Apartado 127, 2781-901 Oeiras, Portugal; 4 Universidade de Lisboa, Faculdade de Ciências, 1749-016 Lisboa, Portugal

## Abstract

**Background:**

Nanotechnology has the potential to provide agriculture with new tools that may be used in the rapid detection and molecular treatment of diseases and enhancement of plant ability to absorb nutrients, among others. Data on nanoparticle toxicity in plants is largely heterogeneous with a diversity of physicochemical parameters reported, which difficult generalizations. Here a cell biology approach was used to evaluate the impact of Quantum Dots (QDs) nanocrystals on plant cells, including their effect on cell growth, cell viability, oxidative stress and ROS accumulation, besides their cytomobility.

**Results:**

A plant cell suspension culture of *Medicago sativa *was settled for the assessment of the impact of the addition of mercaptopropanoic acid coated CdSe/ZnS QDs. Cell growth was significantly reduced when 100 mM of mercaptopropanoic acid -QDs was added during the exponential growth phase, with less than 50% of the cells viable 72 hours after mercaptopropanoic acid -QDs addition. They were up taken by *Medicago sativa *cells and accumulated in the cytoplasm and nucleus as revealed by optical thin confocal imaging. As part of the cellular response to internalization, *Medicago sativa *cells were found to increase the production of Reactive Oxygen Species (ROS) in a dose and time dependent manner. Using the fluorescent dye H_2_DCFDA it was observable that mercaptopropanoic acid-QDs concentrations between 5-180 nM led to a progressive and linear increase of ROS accumulation.

**Conclusions:**

Our results showed that the extent of mercaptopropanoic acid coated CdSe/ZnS QDs cytotoxicity in plant cells is dependent upon a number of factors including QDs properties, dose and the environmental conditions of administration and that, for *Medicago sativa *cells, a safe range of 1-5 nM should not be exceeded for biological applications.

## Background

Nanotechnology is a fast-developing industry, having substantial impact on the economy, society and the environment [1] and predictions so far exceed the Industrial Revolution, with a $1 trillion market by 2015 [2]. Nanotechnology has the potential to revolutionize the agricultural and food industry with new tools for the molecular treatment of diseases, rapid disease detection and enhancing plant ability to absorb nutrients. Smart sensors and smart delivery systems will help the agricultural industry to fight viruses and other crop pathogens [3].

However, the novel size-dependent properties of nanomaterials, that make them desirable in technical and commercial uses, also create concerns in terms of environmental and toxicological impact [4].

Nanotoxicology is emerging as an important subdiscipline of nanotechnology and involves the study of the interactions of nanostructures with biological systems. Nanotoxicology aims on elucidating the relationship between the physical and chemical properties of nanostructures with the induction of toxic biological responses [5]. This information is important to characterize nanomaterial in biotechnology, ecosystems, agriculture and biomedical applications [6].

The few studies conducted to date on the effects of nanoparticles on plants have focused mainly on phytotoxicity and how certain plant metabolic functions are affected. The reported effects vary depending on the type of nanoparticle, as well as plant species, and are inconsistent among studies [2]. So far, there is only one report of nanoparticle toxicity in cells of a photosynthetic organism, the green microalgae *Chlamydomonas reinhardtii*, in which the toxicity of two types of widely used nanomaterials (TiO2 and CdTe) was evaluated [7]. No data is available concerning toxicology of Quantum Dots (QDs) in higher plant cells [8].

QDs are inorganic semiconductor nanocrystals, typically composed of a cadmium selenide (CdSe) core and a zinc sulphide (ZnS) shell and whose excitons (excited electron-holepairs) are confined in all three dimensions, giving rise to characteristic fluorescent properties. QDs are extremely photostable, bright and are characterized by broad absorption profiles, high extinction coefficients and narrow and spectrally tunable emission profiles [9].

Cell-based in vitro studies play an essential role on meaningful toxicity testing. They allow the setting up of high-throughput systems for rapid and cost-effective screening of hazards, while targeting the biological responses under highly controlled conditions [4]. The evaluation of five categories of cellular response, including reactive oxygen species (ROS) production and accumulation, cell viability, cell stress, cell morphology, and cell-particle uptake, are central themes in such testing [10].

Aiming to develop a nano-strategy using coated QDs conjugated with specific biomolecules to precociously identify the presence of fungal infections in *Medicago sativa *(a perennial pulse with economic relevance) we established a fine plant cell suspension culture that was subsequently used to investigate the potential cytotoxicity of CdSe/ZnS Mercaptopropanoic acid coated QDs and its uptake at cellular level.

## Methods

### Cell suspension culture establishment

Cell suspension cultures were established from a *Medicago sativa *line M699, seeds being kindly provided by Diego Rubiales (IAS-CSIC, Spain). Well-developed petioles from 25 day old in vitro germinated M699 seedlings were used as explants for callus induction. Petioles were placed in solid Murashige & Skoog (M&S) medium supplemented with 0.5 mg/L of 2.4-D and kinetin and 5 mg/L of dithiothreitol, maintained in growth chamber under a 16 hours photoperiod and a day/night temperature of 24°/22°C (Phytotron Edpa 700, Aralab, Portugal).

Two friable portions of 8 weeks old dark grown callus from petioles were placed in a 250 mL Erlenmeyer flask with 50 mL of liquid M&S medium, supplemented with the same growth hormone composition used for callus phase. The flasks were maintained in an orbital shaker at 110 rpm (Innova 4900, New Brunswick Scientific, Germany) in the dark, at 24°C. After 10 days, 100 mL of fresh medium was added. 10 days later, 100 mL of fresh medium was added to 20 mL of decanted cell suspension culture. Until an adequate cell density was obtained, the cells were pelleted and medium replaced every 8/9 days. When stabilized, suspensions were sub cultured every 8 days transferring 20 mL to 100 mL of fresh medium (in 250 mL Erlenmeyer flasks). Growth regulators were always filter sterilized through 0.2 μm Orange Scientific filters and added to cooled autoclaved medium (20 minutes at 121°C).These cell suspension cultures will be referred to as stock.

### Synthesis, solubilisation and characterization of CdSe/ZnS core shell QDs

All chemicals unless indicated were obtained from Sigma-Aldrich and used as received. UV-vis absorbance spectra were taken using a Beckman DU-70. Photoluminescence spectra were recorded with a SPEX Fluorolog spectrofluorimeter.

TOPO/HDA - capped CdSe nanocrystals were synthesized using standard procedures [11]. This typically generates CdSe nanocrystals with the first absorption peak around 580-590 nm and a diameter of 3.6-4.5 nm. For the synthesis of core-shell CdSe/ZnS QDs, the cores obtained were monodisperse after passivation with at least 5 monolayers of ZnS. Passivation was obtained using the SILAR method [12] that consists of alternating injections of Zn and S precursors to the solution containing CdSe-core nanocrystals suspended in octadecene/hexadecylamine. After extraction with methanol, centrifugation and decantation, the particles were dispersed in chloroform for further processing.

The Mercaptopropanoic acid coated nanocrystals were synthesized by the phase transfer method as described previously [12]. The obtained Mercaptopropanoic acid coated CdSe/ZnS QDs were then concentrated using a Sartorius Vivaspin 6 tube (cutoff 10 KDa) at 7.500 g.

For the characterization of the synthesized CdSe/ZnS QD nanoparticles Transmission Electron Microscopy (TEM) was used. Low resolution images were obtained using a JEOL 200CX traditional TEM operating at an acceleration voltage of 200 kV.

Quantum yields were measured relative to Rhodamine 6G with excitation at 530 nm. Solutions of QDs in chloroform or water and dye in ethanol were optically matched at the excitation wavelength (λ = 530 nm).

Dynamic Light Scattering (DLS) analysis was performed using a Nano series dynamic light scatterer from Malvern. With this equipment the hydrodynamic diameter (HD) and zeta potential (ξ) of synthetic CdSe/ZnS and their corresponding Mercaptopropanoic acid coated particles were measured. For the HD analyses all the samples were between 0.06 and 0.3 μM and filtered through a 0.2 μm filter before analyses. HD were obtained from number-weighted size distribution analysis and reported as the mean of triplicate measurements. ξ-Potential for Mercaptopropanoic acid coated QDs with concentration between 0.06 and 0.3 μM were measured in H_2_O Milli-Q basified to pH = 12. Values are reported as the average of triplicate runs consisting of 20 runs at 25°C. Similarly, the ξ-potential of the mercaptopropanoic acid-QDs in M&S medium was measured using the DLS equipment. Value reported is the average of triplicate runs consisting of 14 runs at 25°C.

### Imaging and Microscopy settings

Unless stated otherwise, images were acquired in a Nikon Eclipse TE2000-S (Japan) inverted microscope equipped with a HMX-4 100 W Mercury lamp and appropriate filter settings. Images were acquired with an Evolution MP 5.1 megapixel digital CCD Color Camera (Media Cybernetics) controlled by Image Pro Plus 5.0 software (Media Cybernetics).

Thin time-course confocal optical sections (~2 μm thick) were acquired with a Leica SP-E Confocal Laser Scanning Microscope using <20% laser intensity and operating in the mode 1024 × 1024, 400 Hz (~1/2 sec per frame). A × 20 Plan Apo dry objective (NA = 0.75) (Leica) was used. For quantification purposes, gain and offset settings were kept constant so that the average background pixel intensity was between 0 and 10 and the fluorescent signal coming from the cells was between 60 and 220 (0-255 scale for 8 bit images).

### Effect of Mercaptopropanoic acid-QDs supplement on the plant cell growth

A 250 mL Erlenmeyer flask with 120 mL of 8 days old *M. sativa *cell suspension culture was randomly taken from the stock and 4 mL aliquots were inoculated in five 100 mL Erlenmeyer flaks containing 20 mL of fresh medium. Three days after sub-culture a suspension of mercaptopropanoic acid -QDs was added to two of the five Erlenmeyer flasks in order to obtain the final concentration of 100 nM in the culture medium. At day 8 of culture they were sub-cultured by gentle setting and addition of 20 mL of fresh medium. Then, every day, and during 8 days, small aliquots (about 0.5 mL) of the five Erlenmeyers were taken and cells were counted (three counts per aliquot) in a Neubauer Chamber. This allowed to establish a growth curve for the *M. sativa *cells culture.

### Effect of Mercaptopropanoic acid-QDs supplement on the plant cell viability

Cell viability was assessed through the activity of cytoplasmic esterases that hydrolyze FDA to yield the fluorescent product fluorescein, which accumulates intracellularly if the cell membrane remains functional [13].

A 250 mL Erlenmeyer flask with 120 mL of a 8 days old *M. sativa *cell suspension culture was randomly taken from the stock and 2 mL aliquots were inoculated in five 50 mL Erlenmeyer flaks containing 10 mL of fresh medium. After 3 days of subculture a suspension of mercaptopropanoic acid -QDs was added to two Erlenmeyer flasks in order to obtain the final concentration of 100 nM in the culture medium. Before adding the mercaptopropanoic Acid-QDs and every day after, viability of the cultures was checked using the Fluorescein Diacetate (FDA) method [14].

A stock solution of FDA was prepared by adding 10 mg of FDA and 2 mL of acetone and kept at -20°C. A diluted solution of FDA was then freshly prepared at the time of the assay, adding 20 μL of the stock solution to 1 mL of MS medium. Finally, a drop of both suspension culture and diluted FDA was placed on a microscope slide. The counts were performed using an inverted microscope, with excitation at 488 nm. Two preparations were made from each Erlenmeyer and the number of viable cells was counted in five random spots per microscope slide.

### QDs uptake by the plant cells

A 250 mL Erlenmeyer flask with 120 mL of 4 days old *M. sativa *suspension culture was randomly taken from the stock. From this flask, 2 mL aliquots were placed in 50 mL Erlenmeyer flasks (in triplicate), and a volume of mercaptopropanoic acid -QDs to obtain the final concentration of 10 nM was added. After 48 hours samples were visualized using a confocal and an inverted microscope.

### Effect of mercaptopropanoic acid-QDs supplement on ROS accumulation and oxidative stress

ROS formation in cultures exposed to the mercaptopropanoic acid-QDs was evaluated using three different probes: 3,3'-diaminobenzidine (DAB) and nitroblue tetrazolium (NBT) that are specific to hydrogen peroxide (H_2_O_2) _and superoxide anion (O_2_^- ^·) respectively, and 2',7'- Dichlorodihydrofluorescein diacetate (H_2_DCFDA) that is a nonspecific probe for ROS accumulation.

All assays were carried out in 6 well plates (Orange Scientific) to reduce the experimental volume and subsequently the amount of mercaptopropanoic acid-QDs required. This also allowed performing the assays in a standard and randomized approach.

For each experiment two controls were prepared. A negative control was used consisting of cells placed in the same conditions as the assay but without the addition of mercaptopropanoic acid-QDs. As a positive control, cells were heated at 45°C during 20 minutes.

#### 1) H_2_O_2 _detection

Production of H_2_O_2 _at the cellular level was examined by applying the (DAB) staining technique described by Thordal-Christensen *et al. *[15], with few modifications. DAB reacts rapidly with H_2_O_2 _in the presence of peroxidase, forming a brown polymerized product.

A 250 ml flask with 120 mL of 7 days old cell suspension culture of *M. sativa *was randomly taken from the stock to establish the following experimental setup: 2 mL of suspension culture plus 0.1 mg/mL of DAB (negative control), 2 mL of suspension culture treated 45°C for 20 minutes plus 0.1 mg/mL of DAB (positive control), 2 mL of suspension culture plus 10 mM of H_2_O_2 _plus 0.1 mg/mL of DAB and 2 mL of suspension culture plus 32.6 μL of mercaptopropanoic acid-QDs to a final concentration 100 nM plus 0.1 mg/mL of DAB. All samples were placed in sterilized 6 well plates (in triplicate), in an orbital shaker at 110 rpm, in the dark, at 24°C. After 1 hour and half, samples were taken from each assay and observed using an inverted microscope.

#### 2) O_2_^- ^• detection

The detection of O_2_^- ^• was carried out as described by Fryer *et al. *[16] with slight modifications, and similarly to the DAB assay.

Nitro-substituted aromatics such as nitroblue tetrazolium can be reduced by O_2_^-^• to the monoformazan (NBT^+^) [17,18], with the accumulation of dark spots of blue formazan [19].

A 250 ml flask with 120 mL of 7 days old cell suspension culture of *M. sativa *was randomly taken from the stock to establish the following experimental setup: 2 mL of suspension culture with a final concentration of NBT of 60 nM (negative control), 2 mL of suspension culture treated 45°C for 20 minutes with a final concentration of NBT of 60 nM (positive control), 2 mL of suspension culture plus 32.6 μL of mercaptopropanoic acid-QDs to a final concentration of 100 nM plus a final concentration of NBT of 60 nM. All samples were placed in sterilized 6 well plates (in triplicate), in the orbital shaker at 110 rpm, in the dark, at 24°C. After 4 hours samples were visualized using an inverted microscope in bright field.

#### 3) Cellular oxidative stress assay

2',7'- Dichlorodihydrofluorescein diacetate (H_2_DCFDA) was used to determine cellular oxidative stress as described by Ortega-Villasante *et al. *[20], using the same procedure as for the previous assay, but adding 5 μM of H_2_DCFDA instead of NBT. After 1 hour and half samples were visualized using an inverted microscope (λ_ex _= 488 and λ_em _= 525 nm).

H_2_DCFDA diffuses passively through the cellular membrane and then is enzymatically hydrolysed by intracellular esterases to 2',7'-dichlorodihydrofluorescein (DCFH). This nonfluorescent product is converted by ROS into DCF (2',7'-dichlorofluorescein), which can easily be visualized by a strong fluorescence around 525 nm when excited at 488 nm.

The NBT and H_2_DCFDA assays were repeated for longer periods of exposure of cell suspension cultures to the mercaptopropanoic acid-QDs. A 250 mL flask with 120 mL of 8 days old cell suspension culture was randomly taken from the stock, 4 mL and 20 mL aliquots were inoculated in a 100 mL and 250 mL Erlenmeyer flask containing 20 mL and 100 mL of fresh medium, respectively. Three days after subculture a suspension of mercaptopropanoic acid -QDs to obtain the final concentration of 100 nM in the culture medium was added to the Erlenmeyer flask containing 24 mL of cell suspension culture. Finally, on 5^th ^and 6^th ^day the same experimental protocol described above for the two probes was applied.

#### 4) Oxidative stress dose response assay

A 250 mL flask with 120 mL of 3 days old cell suspension culture was randomly taken from the stock. 2 mL (in triplicate) were placed in sterilized 6 well plates. Aliquots of mercaptopropanoic acid -QDs were added to each well to obtain the following final concentrations: 1 nM, 5 nM, 10 nM, 20 nM, 40 nM, 60 nM, 100 nM, 120 nM and 180 nM. All plates were placed on an orbital shaker at 110 rpm in the dark at 24°C. After 48 hours of incubation, an aliquot of H_2_DCFDA, to obtain a final concentration of 5 μM, was added to all treatments including to the negative and positive (cells heat treated 45°C for 20 minutes) controls. After 1 hour, samples from each treatment were visualized using an inverted microscope and the fluorescence was quantified in terms of average pixel intensity, using the commercial program ImageJ. Images were acquired 1 hour after the H_2_DCFDA addition, always with the same settings and exposure time.

### Statistical analysis

All results are presented as the mean ± standard deviation (SD). One-way ANOVA was used to test for significant differences among average fluorescence intensity (Microsoft Office Excel 2007). The result was considered significant if p < 0.001, when compared to the control.

## Results and Discussion

### mercaptopropanoic acid-QD stability

**Figure 1:**
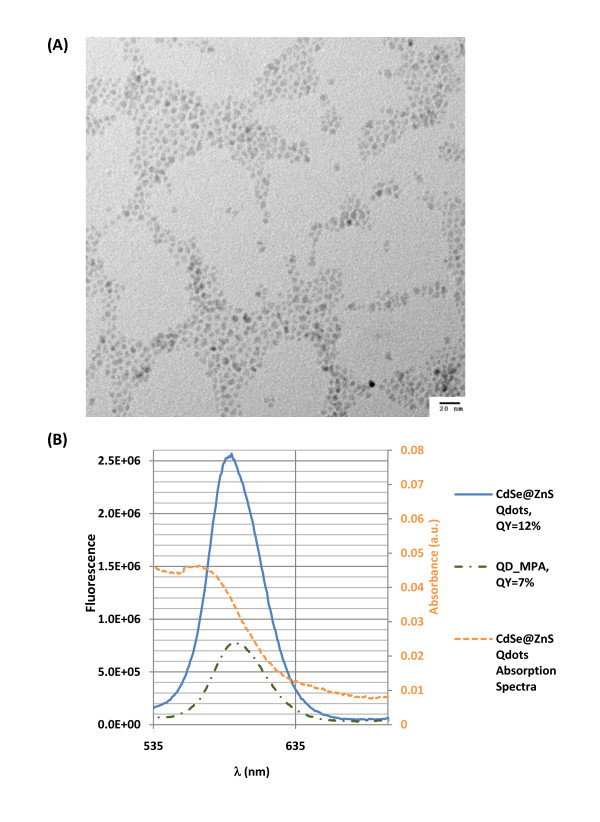
**Characterization of the synthesized CdSe/ZnS QD nanoparticles**. (A) TEM image (acceleration voltage of 200 kV) of CdSe cores after 5 monolayer's of ZnS. (B) Emission spectra and quantum yields for synthetic CdSe/ZnS and their corresponding hydrophilic QDs. The orange spectra is an example of UV-Vis spectra with abs = 0,05 at the excitation wavelength (530 nm).

The mercaptopropanoic acid -QDs used in this work had a size distribution between 4 and 6 nm (Fig. 1a) and a quantum yield of 7% (relative to Rhodamine 6G) (Fig. 1b).

The properties of nanomaterials can change, sometimes dramatically, when placed in contact with biological systems [21]. Nanoparticle aggregation influences their uptake by cells, and variables such as the surface ligands and the solution composition influence nanoparticle suspension stability [22]. Assessment of mercaptopropanoic acid-QD stability and aggregation under the assay conditions was then necessary to correctly interpret their biological effects.

Mercaptopropanoic acid-QDs were supplied in water with a zeta (ζ) potential of -45,6 mV (Table 1), but introduction into the M&S culture medium decreased the ζ and the mercaptopropanoic acid-QDs tended to aggregate, particularly when applied to the culture at relatively high concentrations. The ζ potential of the culture medium was -12.5 mV, well under the absolute value of [25 mV], above which the system is considered to be in a disperse state.

**Table 1:** Results of size and zeta potential measurements for synthetic CdSe/ZnS and their corresponding hydrophilic QDs in water.

Sample	Hydrodynamic diameter (nm)	ξ-Potential (mV)
**CdSe/ZnS (CHCl_3_)**	9,3	-

**MPA- QD (H_2_O)**	13,5	-45,6

The QD surface coating is also considered a critical factor for the extent and time scale of their potential cytotoxicity. Mercaptopropanoic acid is known to be one of the smallest ligands among deprotonated thiols (thiolates) most often used to stabilize QDs in solution [23].

### Effect of mercaptopropanoic acid-QDs on cell growth

The mercaptopropanoic acid-QD concentration used in this study (100 nM) was one order of magnitude higher than those often referred to in the literature [24,25]. It was adopted to ensure that an impact on cell viability could be detected since experiments with lower concentrations did not reveal any consistent effects (data not shown).

**Figure 2:**
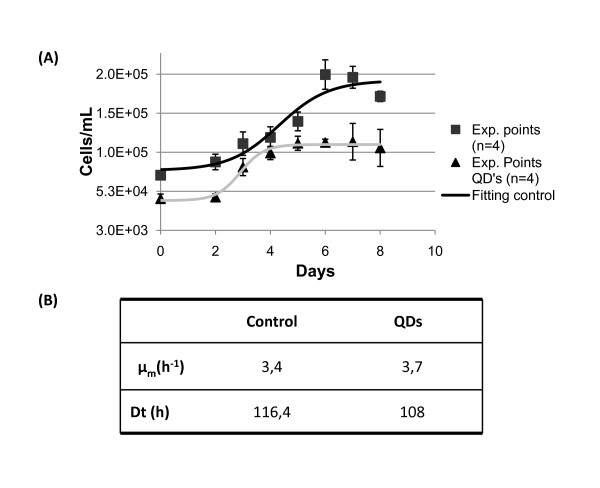
**Effect of mercaptopropanoic acid-QDs in *Medicago sativa *cell growth and kinetic parameters**. (A) Growth curve. Black line refers to control cultures and grey line to cultures treated with the 100 nM of QDs. The fitting was obtained using the software TableCurve 2 D. Error bars represent standard deviation. (B) Maximum specific growth rate (μ) and duplication time (Dt) of control suspension cultures and suspension cultures treated with QDs.

At the end of the cell cycle (8 days), cell suspensions grown in the presence of mercaptopropanoic acid-QDs showed a reduced biomass production compared to the control and all isolated cells were plasmolyzed (data not shown). However, when cells grown with or without the mercaptopropanoic acid-QDs, were sub-cultured, all the suspensions (test and control) showed similar growth parameters (Fig. 2b). Both cultures had comparable growth profiles (Fig. 2a) but those previously treated with mercaptopropanoic acid-QDs started the exponential growth phase one day sooner and reached the stationary phase earlier.

These results suggest that cultures recovered from QD damage in the second cell growth cycle which could be explained by the observed cell aggregation after the addition of the mercaptopropanoic acid-QDs. Cell aggregation can be interpreted as a response by plant cells to the presence of mercaptopropanoic acid-QDs, which may induce cell wall lignification and crosslinking of cell wall components. Cell aggregation was also reported in *Chlamydomonas reinhardtii *cultures in the presence of CdTe and TiO_2 _nanoparticles [7]. Cellular aggregation will provide greater protection against toxic agents, particularly for cells in the middle of the aggregates,. These cells will be able to act has a viable *inoculum *when subcultured in fresh culture medium.

### Effect of mercaptopropanoic acid-QDs on cell viability

**Figure 3:**
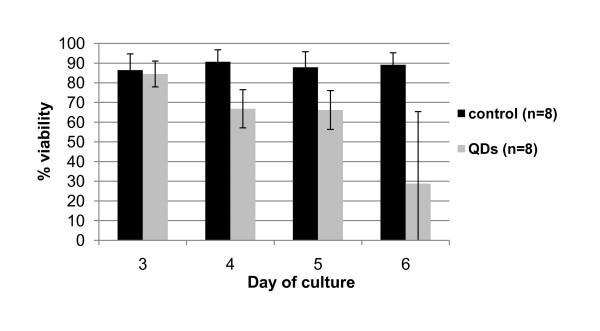
**Effect of 100 nM of mercaptopropanoic acid-QDs on cell viability determined with FDA method**. Black line refers to control cultures and grey line to cultures treated with the QDs. Day 3 counts were performed before the QD addition. Error bars represent standard deviation.

Twenty four hours after the exposure to 100 nM of mercaptopropanoic acid -QDs, cell viability had already been reduced by 6% (Fig. 3).

After 72 hours in contact with mercaptopropanoic acid-QDs, isolated cells, or cells in small aggregates, were not viable and most of them were plasmolysed. Less than 50% of the isolated cells were viable when compared with the control. However, most cells were found in large clusters. These clusters seemed to be viable, despite the significant decrease in fluorescence intensity when compared to the control (data not shown). This explains why subcultures previously subjected to mercaptopropanoic acid-QDs continued to grow. Cell aggregation seemed to guarantee in this case a certain level of protection against toxicity probably due to a certain impermeabilization of the cell wall in response to the imposed stress, as suggested previously.

Envisaging the application of this type of mercaptopropanoic acid-QDs to plant organs our results showed that elevated concentrations of mercaptopropanoic acid-QDs reduced significantly plant cell viability, mainly in cases where cells were isolated. They also showed that some cells in aggregates maintain a certain degree of viability.

### Quantum dot uptake

**Figure 4:**
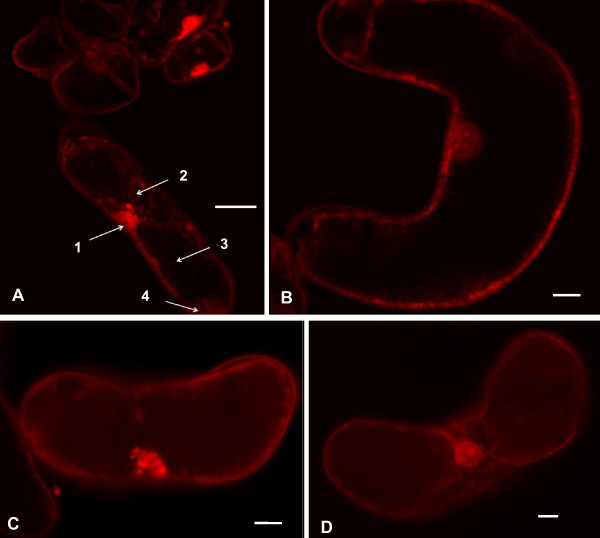
**Confocal (A, B) and wide field (C, D) fluorescence images of *M. sativa *cells after 48 hours of incubation with 10 nM of mercaptopropanoic acid-QDs showing QD internalization**. Arrows point to 1- nucleus, 2- cytoplasmic strands, 3-vacuole and 4-hyaloplasm. Scale bar = 20 μM.

*M. sativa *cells internalized mercaptopropanoic acid-QDs as shown in Fig. 4a and 4b. Fluorescent spots due to mercaptopropanoic acid-QDs could be observed mainly in the nucleus and in the cytosol. Also cytoplasmic strands presented fluorescent particles, the vacuole, which occupies the majority of the cell volume, being the exception.

Images acquired using the inverted microscope (Fig. 4c and 4d) clearly showed the fluorescence of mercaptopropanoic acid-QDs in several structures, such as the nucleus and nucleolus, the cytoplasm and cytoplasmic strands. In a recent study on the biogenic uptake of platinum (Pt), the roots of *Medicago sativa *were found to accumulate Pt *in vivo *in the form of nanoparticles on cell walls and organelles [24].

When the putative cytotoxicity of nanoparticles is determined, it is prudent to consider whether cells do in fact encounter individual nanoparticles, in contrast to aggregates of several nanoparticles; this becomes particularly relevant when studying internalization. Therefore it is necessary to have in mind that if the nanoparticles had not aggregated in the culture medium, probably more cells would internalize them.

### ROS accumulation and oxidative stress

#### 1) H_2_O_2 _detection

**Figure 5:**
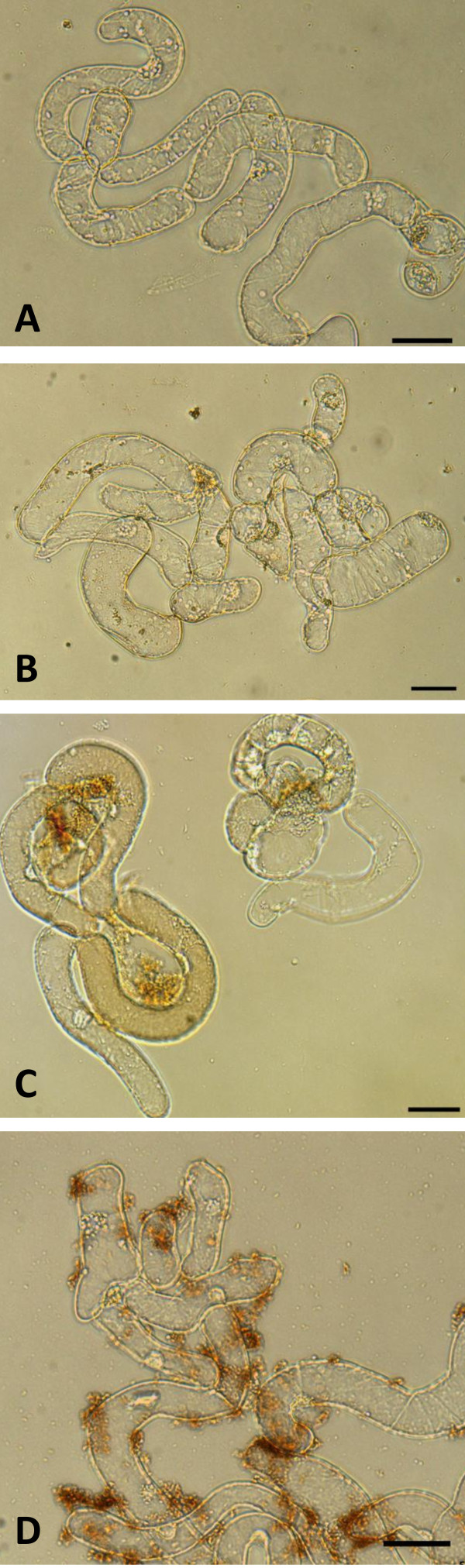
**Cell suspension culture controls for the DAB assay**. (A) Without treatment. (B) Treated with DAB (negative control). (C) Heat-treated (45°C for 20 minutes) to induce the production of H_2_O_2 _(positive control). (D) Treated with 10 mM H_2_O_2 _and DAB to visualize the peroxidase activity. All experiments were visualized 1 hour and half after the addition of DAB, in bright field. Scale bar = 50 μM.

Cells treated with DAB (Fig. 5b), in the absence of any oxidative stress, did not present the typical brown precipitate. Cells heat treated for 20 min at 45°C with subsequent addition of DAB (Fig. 5c), showed a brownish colour as well as precipitates indicative of oxidative stress due to H_2_O_2 _presence. Cells treated with 10 mM of H_2_O_2, _followed by the addition of DAB, showed the same brown precipitate (Fig. 5d) confirming that the cells were able to metabolize H_2_O_2_.

**Figure 6:**
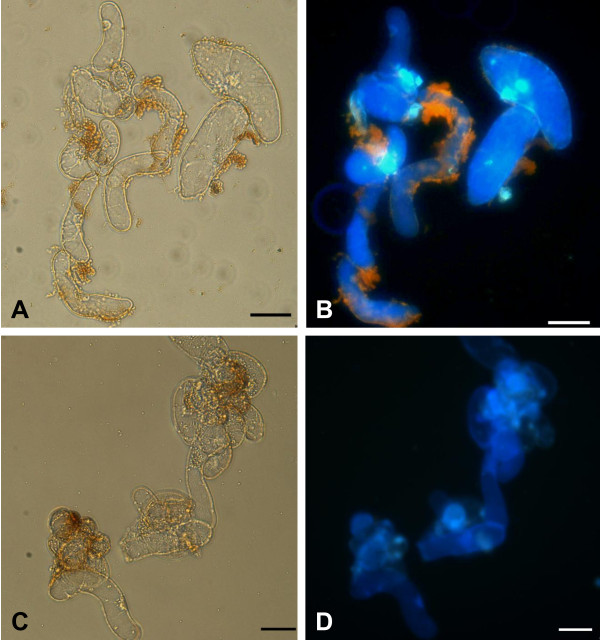
**DAB assay for H_2_O_2 _detection**. (A, B) Cell suspension culture treated with 100 nM of QDs plus DAB. (C,D) Cell suspension culture treated only with DAB. The orange/brownish precipitates around cells are QDs and not due to the DAB polymerization as revealed by UV light excitation. Scale bar = 50 μM.

The addition of 100 nM of mercaptopropanoic acid-QDs to *M. sativa *cells did not seem to induce H_2_O_2 _production, as illustrated in Fig. 6. The resulting orange/brownish coloration around the cells was due to precipitated mercaptopropanoic acid -QDs, which was confirmed by comparing the UV light picture (upper) with the UV image of cells and DAB (lower) where no orange coloured spots were detected.

The comparison of the cells reaction in the presence of mercaptopropanoic acid-QDs with the reaction when H_2_O_2 _was added to the culture, leaded to the conclusion that the production of H_2_O_2 _induced by the addition of mercaptopropanoic acid-QDs is far less than 10 mM, if any.

#### 2) O_2_^- ^• detection

**Figure 7:**
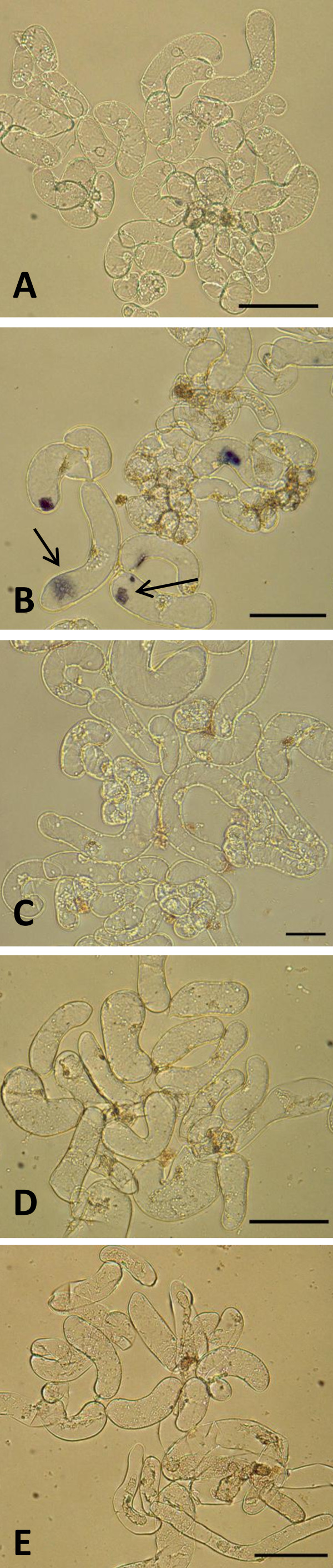
**Detection of O_2_^- ^• by the NBT assay in cell suspension cultures**. (A) Treated with NBT. (B) Treated 20 minutes at 45°C and NBT exhibiting blue formazan spots due to O_2_^- ^• production (indicated by arrows). (C) Treated with 100 nM QDs and stained with NBT showing no blue formazan spots. (D) Treated with 100 nM QDs for 48 hours and NBT and (E) treated with 100 nM QDs for 72 hours and NBT confirming that there is no O_2_^- ^• production. All samples were visualized 4 hours after the NBT addition in bright field. Scale bar = 50 μM.

After heating at 45°C for 20 minutes, cells exhibited dark blue formazan spots (Fig. 7b). These deposits indicated that O_2_^- ^• was produced by the cells in response to heat stress, since no spots were seen in the controls (Fig. 7a). The presence of formazan deposits indicate that in these cells the rate of O_2_^- ^• production had become significantly greater than the rate of detoxification [16].

Studies that applied the NBT technique were mainly focused on evaluating responses in plant tissues rather than in single cells. The few reports in cell suspension cultures that used this technique quantified the response spectrophotometrically, rather than study the precise location of the formazan deposits [26,19].

In *M. sativa *cell suspension cultures treated with mercaptopropanoic acid-QDs, no blue formazan spots were detected, as shown in Fig. 7c, which could suggest that the interaction of 100 nM of mercaptopropanoic acid-QDs did not induce the formation of O_2_^- ^• after 4 hours of exposure. Also cultures exposed to mercaptopropanoic acid-QDs for 48 and 72 hours (Fig. 7d and 7e respectively) did not show any noticeable production of O_2_^- ^• since no blue formazan spots were detected.

A previous study [27] on the generation of free radicals (in an aqueous, cell-free system) by three types of mercaptopropanoic acid-QDs revealed that while CdS QDs apparently had sufficient redox power to generate hydroxyl and superoxide radicals, CdSe QDs exclusively generate hydroxyl radicals.

Chloroplasts are a major site for ROS generation in plants due to the photosynthesis process. Peroxisomes and glyoxysomes are other major sites of ROS generation in plants during photorespiration and fatty acid oxidation, respectively [28]. However plant cell cultures used in this work are dark grown and therefore do not have differentiated chloroplasts. This also may explain why no O_2_^-^• was found in our system.

#### 3) Cellular oxidative stress assay

**Figure 8:**
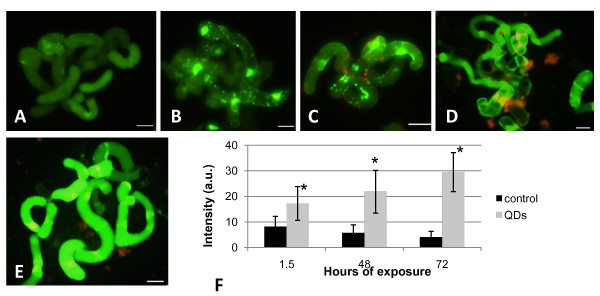
**General oxidative stress assay in cell suspension cultures**. (A) Treated with H_2_DCFDA showing a basal level of fluorescence. (B) Treated 20 minutes at 45°C and H_2_DCFDA exhibiting a fluorescence increase due to an oxidative stress. (C) Treated with 100 nM QDs and H_2_DCFDA. (D) Treated with 100 nM QDs for 48 hours and H_2_DCFDA and (D) treated with 100 nM QDs for 72 hours and H_2_DCFDA. Scale bar = 50 μM. (F) graphic representation of mean fluorescence intensity in A-E. Black bars represent the mean of pictures from control cultures (cells + H_2_DCFDA) and grey bars the QD treated cultures in the three exposure times performed. Data represent mean from one experiment (n = 6) + SD. Columns with * indicate a significant difference from the control value with *p *< 0.001 (ANOVA).

When treated with H_2_DCFDA, control cells showed a basal-level fluorescent signal (Fig. 8a) that accumulated uniformly in the cytoplasm. This has been also reported in tobacco cells by Ashtamker *et al. *[29]. In the heat treated (45°C/20 min) cell suspension cultures, the DCF signal was significantly increased (Fig. 8b) showing a response in terms of ROS accumulation, also observed in the mercaptopropanoic acid-QDs treated cell suspension cultures (Fig. 8c). These results confirmed the induction of an oxidative stress in cell suspension cultures treated with 100 nM of mercaptopropanoic acid-QDs during 1.5 hours.

To evaluate the response of the cell suspension cultures when exposed to mercaptopropanoic acid-QDs over a longer period of time, QDs addition was performed at day 3 of culture and the H_2_DCFDA assay performed after 48 and 72 hours (Fig. 8d and 8e respectively). A progression of the intensity of the DCF signal could be seen in the cell cultures exposed to mercaptopropanoic acid-QDs, when compared with the controls.

To further clarify the results in terms of the mean fluorescence intensity of DCF, a normalized graphic representation was obtained (Fig. 8f). Comparing the results of the three intensity values obtained over time, it can be concluded that while the control (cells plus H_2_DCFDA) maintained an intensity bellow 8 a.u., cells treated with mercaptopropanoic acid-QDs progressively increased the fluorescence intensity with the exposure time. mercaptopropanoic acid-QD treated cultures increased ROS production in 52%, 73% and 85%, respectively 1.5, 48 and 72 hours after the mercaptopropanoic acid-QD addition, showing that ROS production was intensified in a time dependent manner.

Conclusions about the specificity of H_2_DCFDA are contradictory. DCFH oxidation to DCF can occur as a result of interaction with either H_2_O_2 _or •OH^- ^[30]. Myhrea *et al. *[31] suggest that H_2_DCFDA is sensitive to the oxidation by ONOO^- ^(peroxynitrite, often included as ROS), H_2_O_2 _(in combination with cellular peroxidases), peroxidases alone, and •OH^-^, but not suitable to detect NO, HOCl, and O_2_^- ^• radicals in biological systems. Due to the indiscriminate nature of DCFH, the increase of intracellular DCF fluorescence may not necessarily reflect the levels of ROS directly, but rather an overall oxidative stress index in cells [32,33].

#### 4) Oxidative stress dose response

**Figure 9:**
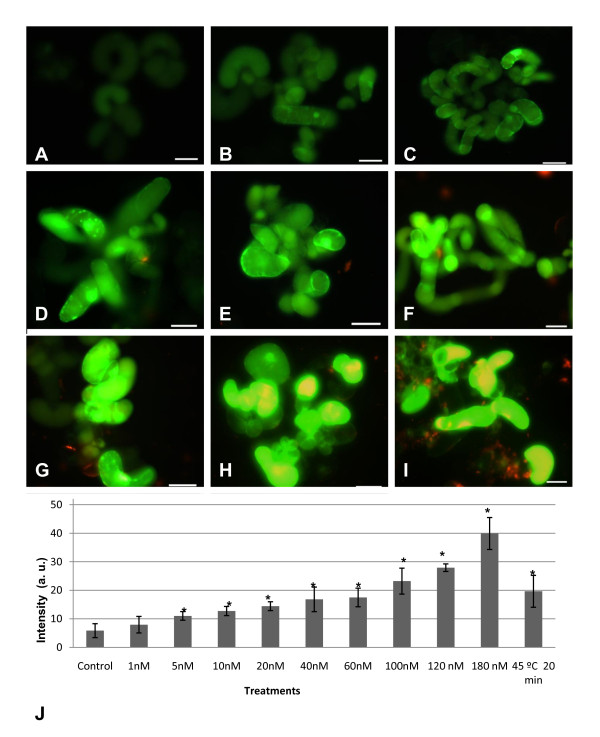
**Oxidative stress dose response assay**. Cell suspension cultures treated with QDs and H_2_DCFDA: (A) 1 nM QDs, (B) 5 nM QDs, (C) 10 nM QDs, (D) 20 nM QDs, (E) 40 nM QDs, (F) 60 nM QDs, (G) 100 nM QDs, (H) 120 nM QDs, (I) 180 nM QDs. Scale bar = 50 μM. (J) graphic representation of mean fluorescence intensity of pictures from cultures subjected to the different treatments. Data representing means from two independent experiments + SD (n = 6 in each experiment), except for the conditions 120 nM and 180 nM which represent one experiment (n = 7). Columns with * indicate a significant difference from the control value with *p *< 0.001 (ANOVA). Fluorescence intensity in cultures treated with QDs shows a linear behaviour represented by Y = 0,1581x + 9,6342 and R² = 0,9735. The H_2_DCFDA assay was performed 48 hours after the QD addition.

Since it is clear that the presence of mercaptopropanoic acid-QDs in cell suspension cultures induces an oxidative stress, it is important to clarify if there is a relation between this cell response and the concentration of mercaptopropanoic acid-QDs. Our results showed that fluorescence increased with the increasing concentrations of mercaptopropanoic acid-QDs (Fig. 9a-i).

The mean fluorescence intensity of the cells subjected to the different QD concentrations was calculated, showing a linear increase (Fig. 9j). Regarding the range of concentrations used, it seems that to have a minor impact in terms of oxidative stress, the concentration range of mercaptopropanoic acid-QDs to be used should be between 1-5 nM, since cultures subjected to this concentration range presented a fluorescence intensity value that was not significantly different from the control. Concentrations above 5 nM led to the intensification of ROS production in more than 47%, measured in terms of fluorescence intensity, with a maximum of 85% at a concentration of 180 nM of mercaptopropanoic acid-QDs.

Heterogeneity in cellular ROS production was also found to accompany the mercaptopropanoic acid-QD concentration which is likely to reflect variability in the physiological conditions of the cells.

## Conclusions

Here we report for the first time the response of *in vitro *cultured *Medicago sativa *plant cells when exposed to Quantum dots. Our results showed that concentrations above 1 nM of mercaptopropanoic acid -CdSe/ZnS QDs induce a cyto-oxidative response in the plant cells.

Plant cells treated with 100 nM of mercaptopropanoic acid-QDs showed a low cytotoxicity in short term exposure (24 hours) with a reduction of 6% in cell viability, which is probably related with the increase of 50% in ROS production. 72 Hours after the QD addition, less than 50% of the isolated cells were viable, when compared with the cells in the control, and ROS production was intensified in 85%.

Plant cells were found to increase the production of ROS in a dose dependent manner, and we shown that concentrations of 1-5 nM could be cyto-compatible for this type of QD. We also concluded that the superoxide anion is not the reactive species involved in the oxidative stress, since no superoxide anion production was detected upon 78 hours of exposure.

Mercaptopropanoic acid -CdSe/ZnS QDs were taken up by the cells of *Medicago sativa *and found to accumulate in the nucleus and the cytoplasm, but not in the vacuoles. This uptake may explain the verified cyto-oxidative stress of QD treated cultures after only 1 hour of exposure.

Together these results confirm that the extent of QD cytotoxicity in plant cell cultures is dependent upon a number of factors including QD properties such as the coated material and surface chemistry, but also depends on the dose and on the environment where they are administered.

The intracellular formation of ROS is believed to be one of the causal factors of QD induced cytotoxicity [10]. Although mercaptopropanoic acid coated CdSe/ZnS QDs are not completely innocuous to plant cells, a safe range of concentrations for biological application can be defined. The results presented in this work contribute to the characterization of the parameters that regulate the toxicity of QDs in plant cell cultures, providing a basis for further work towards the improvement of nanoparticles functionalization and surface-coating strategies.

## List of abbreviations

QDs: Quantum Dots; ROS: Reactive Oxygen Species; Mercaptopropanoic acid-Mercaptopropanoic acid; M&S: Murashige & Skoog; 2.4 D: Dichlorophenoxyacetic acid; FDA: Fluorescein diacetate; DAB: 3,3'-diaminobenzidine; NBT: Nitroblue tetrazolium; H_2_DCFDA: 2',7'-dichlorodihydrofluorescein diacetate.

## Competing interests

The authors declare that they have no competing interests.

## Authors' contributions

ARS performed the majority of the experiments and wrote the first draft of the manuscript. ASM synthesized and characterized the quantum dots and wrote that part of the manuscript. LT contributed to the cell suspension culture establishment. RM contributed to the confocal and inverted microscopy and image processing. CM supervised the QD synthesis and contributed to the elaboration of the manuscript. MCVP contributed to the design of the project. PF participated in the design and coordination of the study, contributed to the interpretation of data and drafting of the manuscript. AO conceived the overall project and participated in the design of the work. All authors read, participated in the writing of the manuscript and approved the final manuscript.
